# Targeting AD and its comorbidities with combination therapies

**DOI:** 10.1002/alz.083429

**Published:** 2025-01-09

**Authors:** Teresa Leon

**Affiliations:** ^1^ Novo Nordisk A/S, Søborg, 2860 Søborg Denmark

## Abstract

Alzheimer’s disease (AD) is a complex disease that is often accompanied by a range of comorbidities, such as cardiovascular disease, diabetes, and depression. These comorbidities can impact the progression of AD and can complicate treatment strategies. Targeting comorbidities in Alzheimer’s disease and developing combination therapies are emerging areas of research.

The following topics will be addressed:

• Review of main AD comorbidities

• Contributors to the pathophysiology and progression of Alzheimer’s disease (i. e neuroinflammation, vascular damage, etc) are common ground in some of the AD comorbidities

• Cognitive function can be impacted by the associated comorbidities (i.e depression and anxiety) and concomitant treatments (i.e sedatives)

• Developing combination therapies that target multiple aspects of Alzheimer’s disease and its comorbidities could prove more effective than targeting a single aspect of the disease

By also targeting comorbidities in Alzheimer’s disease and developing combination therapies, researchers and healthcare providers can find more effective ways to address the complex nature of Alzheimer´s. These strategies may ultimately lead to improve outcomes for patients with Alzheimer’s disease and its comorbidities.

